# The importance of peer review to International Brazilian Journal of Urology

**DOI:** 10.1590/S1677-5538.IBJU.2022.01.02

**Published:** 2021-12-21

**Affiliations:** 1 Universidade do Estado do Rio de Janeiro - Uerj Unidade de Pesquisa Urogenital Rio de Janeiro RJ Brasil Unidade de Pesquisa Urogenital - Universidade do Estado do Rio de Janeiro - Uerj, Rio de Janeiro, RJ, Brasil; 2 Hospital Federal da Lagoa Serviço de Urologia Rio de Janeiro RJ Brasil Serviço de Urologia, Hospital Federal da Lagoa, Rio de Janeiro, RJ, Brasil

In 2021 the International Brazilian Journal of Urology received the highest impact factor of his history and this fact was possible because the serious peer review process of our Journal (1). In this year we received more than 800 papers. The peer review system is a very difficult process because is completely free. his process depends of the hard work of the experts in several topics reviewers by on the topic (2). The Editor-in-Chief would like to thanks all the reviewers and specially to the Doctors: Alexandre Danilovic (Hospital das Clínicas da Faculdade de Medicina da USP -São Paulo, SP, Brasil); Ralf Anding (University Hospital Friederich Wilhelms, University Bonn, Germany); Andrew J. Combs (Weill Cornell Medical College, New York, USA); Eduardo Mazzucchi (Universidade de São Paulo - USP, SP, Brasil); John Denstedt (Department of Surgery, Division of Urology, Western University, St. Joseph's Health Care, Ontario, Canada); Fernando Korkes (Faculdade de Medicina do ABC, Santo André, SP, Brasil); Leonardo Abreu (Universidade Estácio de Sá -Rio de Janeiro, RJ, Brasil); Ricardo Miyaoka (Univ. Estadual de Campinas – UNICAMP, Campinas, SP, Brasil); Silvio Henrique M. Almeida (Universidade Estadual de Londrina - UEL, Londrina, PR, Brasil) and Ubirajara Barroso Jr. (Escola Bahiana de Medicina e Saúde Pública, Salvador, BA, Brasil) who reviewed more than 3 articles during the year and strictly within the deadline,

Thanks a lot!!!!!
